# Adolescent and parental proxy online record access: analysis of the empirical evidence based on four bioethical principles

**DOI:** 10.1186/s12910-025-01182-9

**Published:** 2025-02-20

**Authors:** Josefin Hagström, Maria Hägglund, Charlotte Blease

**Affiliations:** 1 Participatory eHealth and Health Data Research Group, Department of Women’s and Children’s Health, Uppsala University, Uppsala, Sweden; 2 MedTech Science & Innovation Centre, Uppsala University Hospital, Uppsala, Sweden; 3 Digital Psychiatry, Department of Psychiatry, Beth Israel Deaconess Medical Center, Harvard Medical School, Boston, MA USA

**Keywords:** Electronic health record, Online record access, Patient portal, Open notes, Ethics, Adolescents, Parents, Guardians, Adolescent health

## Abstract

**Background:**

During recent decades, providing patients with access to their electronic health records (EHRs) has advanced in healthcare. In the European Union (EU), the General Data Protection Regulation provides individuals with the right to check their data in registries such as EHRs. A proposal for a European Health Data Space has been launched, which will further strengthen patients’ right to have online access to their EHRs throughout Europe. Against these policy changes, scant attention has been paid to the ethical question about whether adolescents and parents should access the adolescent’s EHR, and if so, under what conditions.

**Methods:**

In this paper, we apply biomedical ethical principles to explore key questions about adolescents’ and parents’ access to adolescents’ EHRs, with the aim of informing future discussions about the development of ethical and policy practice guidelines.

**Results:**

Drawing on current empirical research, we find preliminary evidence that in some contexts, patient online record access (ORA) could help to facilitate autonomy for adolescents and parents as well as offering support in managing appointments and medications. Notably, however, we find contrasting perspectives between adolescents’ and parents’ experienced benefits and healthcare professionals’ (HCPs) perceived potential harm, with the latter worried about decreased documentation quality after access. Concerns about capacity to understand their health information, and increased anxiety among adolescents obstruct the support of adolescent autonomy among parents and HCPs. Still, research is limited, particularly with respect to adolescents’ experiences of reading their EHRs, and differences across settings have not been closely examined.

**Conclusions:**

To advance more comprehensive understanding of the effects of ORA, and to inspire greater attention to, and development of, evidence-informed ethical guidance in this domain of clinical practice, we outline a range of empirical questions regarding adolescents’ and parents’ experiences that now warrant further study.

## Background

As healthcare increasingly prioritizes transparency, more patients are gaining access to their electronic health records (EHRs) via patient portals [1]. Access can include test results, diagnoses, medications, referrals, and the visit notes written by clinicians [2]. In the European Union (EU), the General Data Protection Regulation mandates access for adults to their information in registries and a proposed European Health Data Space [3] states that patients should have online record access (ORA) to their EHRs throughout Europe. However, uncertainty remains about granting ORA to the EHRs of children and adolescents, primarily due to clinician concerns about the potential for harm among this patient population [4, 5].

Currently, addressing these worries, a variety of approaches have been adopted within the EU and globally [1, 6]. Implemented access control practices, for both minors and parents, either rely on pre-defined age-based access limits or access is determined on a case-by-case basis. For example, national ORA systems in Sweden, Norway, and the UK apply a minimum access age for minors (age 16) and an age cut-off for parental access (age 11–13) [6, 7]. After the parent loses access and before minors gain access, minors and parents can receive access by application (UK: by discussion with the HCP). After age 16, parents in Sweden and Norway have no access, while in the UK, continued parental access can be retained if the young person consents or is not considered competent to make a decision about access. Conversely, Finland has adopted a case-by-case system with no minimum access age for minors and parental access depending on HCP assessment and minors’ consent [6]. With the current European Health Data Space proposal, it is suggested that EU member states should “enable guardians to act on behalf of their dependent children”, without further specification [3].

However, ethical concerns about whether adolescents and parents should have access to the EHRs of adolescents have received limited empirical input. For example, in an interview study, Norwegian mental health healthcare professionals (HCPs) working in adolescent healthcare expressed the belief that ORA implementation was a political goal rather than being initiated or needed by HCPs [8]. From a medical ethical standpoint, younger children are considered to lack the competence and capacity for potential autonomous decision-making, which requires parents to be responsible and involved in their care. It appears that, as an extension of this perspective, parental access to minors’ EHRs has often been implemented as a proxy for their information in many countries [1, 6]. Yet, as children grow into adolescence, their capacity for autonomy is considered to increase [9] alongside it, there is an attendant ethical need to afford the growing young person greater personal privacy. Although research on minor patients’ and parents’ ORA experiences is scarce, existing evidence indicates the potential for benefits similar to those observed in adult patients, including increased engagement and empowerment, improved recall, and reduced anxiety [10–13]. Balancing this, HCPs worry about patient confidentiality breaches and safeguarding the privacy of minors and parents when access to the EHR is shared.

## Methods

We situate our discussion of ethical considerations for ORA in the case of adolescents by using biomedical ethical principles [14, 15] (see Table 1). This framework was chosen because it is a well-established model of applied ethics, particularly suited to addressing key considerations in the context of adolescent and parental ORA. The framework acknowledges that perceptions of privacy and autonomy are shaped by diverse cultural, religious, and regional factors, as well as by variations in family dynamics and adolescent development. While subject to contextual adaptation, however, the principles are also understood to be universal. Therefore, by grounding the analysis in principles of biomedical ethics, this approach aims to provide an organized structure for discussions about the range of ethical dilemmas that can emerge in contexts where clinicians and patients access EHRs online.

Although the principle of justice is an important aspect of biomedical ethics, it was excluded in our analysis due to limited empirical research into this issue among young people. Existing research among adults has highlighted how ORA may invite disparities in access owing to digital divides [16], whilst other work has shown potential benefits of access in overcoming communication barriers for marginalized groups [17]. Given these complexities, we prioritized the principles of beneficence, nonmaleficence, autonomy and confidentiality in the present paper, as they directly address the pressing concerns in this context. We acknowledge that justice remains a critical ethical principle, however, particularly when considering systemic issues such as equitable access to healthcare resources and disparities in health outcomes. Future work could robustly integrate the justice principle to develop a comprehensive ethical understanding about ORA.

Relatedly, in this paper we adapt “respect for patient autonomy” to the broader consideration of “respect for persons”. This is because ORA may support adolescents’ developing autonomy while safeguarding their welfare. Under this consideration, the parental role is clarified to focus on how ORA aids parents in fulfilling their responsibilities and protecting their child’s best interests, rather than prioritizing their individual autonomy. Additionally, we recognize that minors’ autonomy is a dynamic and evolving process.

Our focus in what follows, therefore, is to identify the key empirical research pertaining to respect for persons including young patients’ developing patient autonomy, nonmaleficence, beneficence, and privacy and confidentiality [15] among adolescents and parents. We recognize that a variety of empirical evidence is necessary to inform ethical practice and we survey current findings drawing on research among various stakeholders - minors, parents, HCPs, and other experts– and link these to specific ethical principles. We also identify the empirical research questions that may help to resolve uncertainties and strengthen ethical practice in this domain of clinical practice.

Owing to the emergence of this field, the majority of the referenced literature stems from a prior scoping review conducted by the authors, which comprehensively mapped existing research on ORA [18]. This scoping review included studies published from 2007 onward, reflecting the timeline of ORA’s gradual implementation. It is important to note that the choice of timeframe aligns with the incremental adoption of ORA, which began to take shape around 2012 in both the Nordic countries and the United States. This period marks the emergence of ORA as a significant healthcare innovation, coinciding with the digitalization of health records and the growing focus on patient empowerment and transparency in care. By drawing on this curated body of literature, and more recently published papers, the analysis aims to offer a robust foundation for exploring the ethical dimensions of ORA while acknowledging the relatively recent nature of its implementation and the evolving scholarship in this field.

**Fig. 1:**
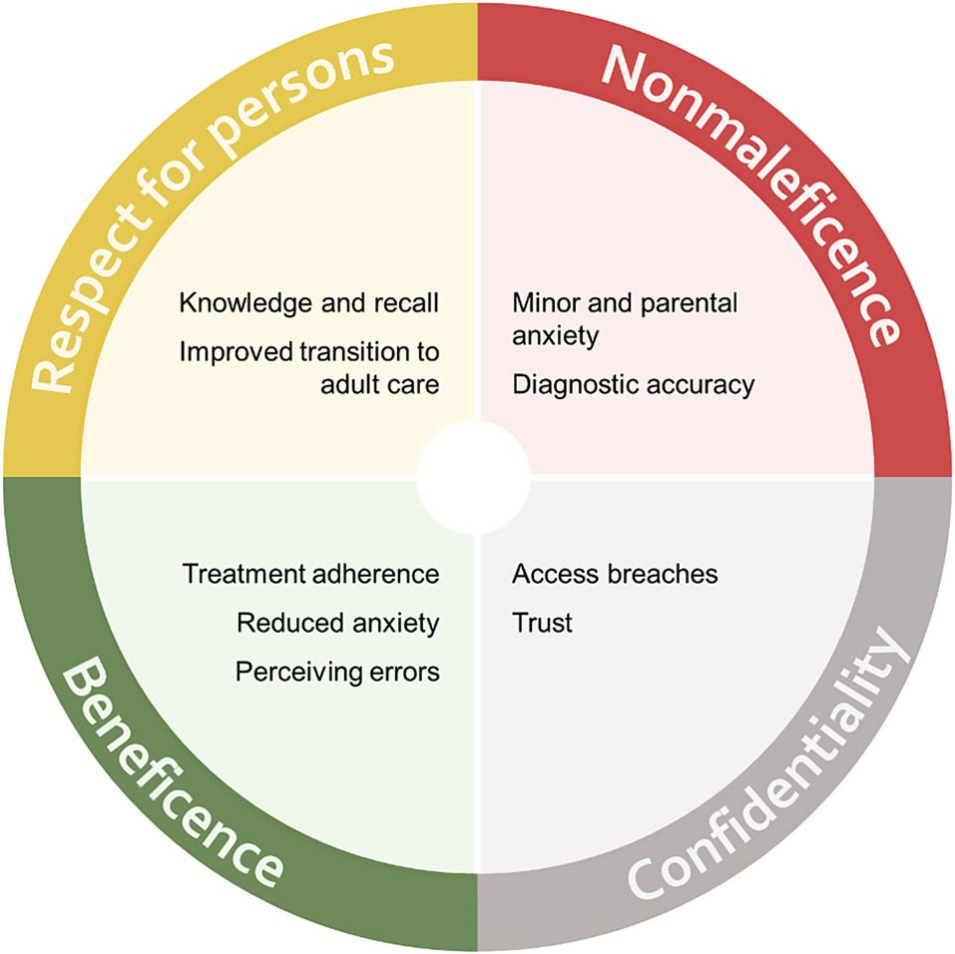
Topics identified related to ORA in paediatric contexts and their link to each biomedical ethical principle included

This paper aims to offer a preliminary exploration about how ethics and empirical research may intersect in this area of innovation. Related to the emerging challenges in his area of research, a word on terminology: In this paper, the terms “minors” and “children” refer to patients of any age under 18, while “adolescents” refers to individuals aged 12–19, or as specified in the text when referring to studies. While this paper focuses on adolescents aged 12–19 years old, ethical issues for minors differ across childhood and adolescence adding further complexities to ethical analyses. An overview of biomedical ethical norms included can be found in Box 1. The issues we have identified as relevant are listed in Fig. 1, related to each biomedical ethical principle.

**Table 1:** Empirical research questions by ethical principles, with suggested topics for future study

	Empirical research questions^a^		Suggested topics for future study
Ethical principle	Does ORA…		
	Adolescents’ access	Parental access	
Respect for Persons	I. Influence adolescents’ perceptions of empowerment? II. Influence understanding about their diagnosis, treatment, or care plan? III. Improve recall among adolescents about the treatment or care plan?	I. Influence parents’ perceptions of empowerment? II. Influence parents’ understanding about their child’s diagnosis, treatment, or care plan? III. Improve parents’ recall about the treatment or care plan?	• Adolescents’ and parents’ comprehension of EHRs • Adolescents’ interest in accessing their EHRs and using patient portals • Adolescents’ and parents’ perceived improvement of knowledge and recall from ORA (using validated or quantitative measures) • The mechanisms of support of the adolescence-adulthood transition
Nonmaleficence	IV. Decrease adolescents’ willingness to seek healthcare? V. Increase adolescents’ and parents’ worry and anxiety? VI. Influence diagnostic accuracy?	IV. Increase parents’ worry and anxiety?	• Adolescents’ and parents’ experiences of distress and anxiety from ORA • Reducing patient anxiety through increased empathy in EHR language, for example using AI-powered tools • Whether ORA changes the reporting of differential diagnosis and/or incurs language changes in the EHR • Whether clinician training in ORA strengthens professional autonomy • HCPs’ attitudes to and experiences of blocking information from adolescents’ EHRs
Beneficence	VII. Increase adolescents’ adherence to medications? VIII. Change attendance rates at visits? IX. Improve adolescents’ wellbeing? X. Influence rates of medication errors?	V. Change attendance rates at visits? VI. Improve parents’ wellbeing? VII. Influence rates of medication errors?	• Whether ORA influences adolescents’ adherence to treatment and medication, as well as in attending appointments • Adolescents’ and parents’ emotional experiences from reading the EHRs, specifically regarding adolescents with serious health conditions • Adolescents’ and parents’ experiences of identifying errors in the EHR, and subsequent actions taken to rectify inaccuracies
Privacy and Confidentiality	XI. Change adolescents' perceptions of privacy about their health information? XII. Lead to breaches of confidentiality? XIII. Improve adolescents’ trust with their healthcare provider? XIV. Influence the relationship between the adolescent, parent, and HCP?	VIII. Lead to breaches of confidentiality? IX. Influence the relationship between the adolescent patient, parent, and HCP?	• What portal structures strengthen perceptions and experiences of confidentiality of adolescents and parents in EHRs • Adolescent patients’ and parents’ experiences of approaches allowing adolescents’ control of their EHRs • Under what clinical contexts, and for which patients, ORA can positively and negatively affect trust among adolescents and parents, and among both respective groups and their HCPs • How adolescents’ perceptions of confidentiality of adolescents affect their decisions to seek care

^a^We recommend that research investigates adolescents’ and parents’ experiences in different contexts and settings. We envisage that a variety of methodologies should be applied to tackle these research questions including mixed methods survey research, natural language processing, and other techniques aimed at understanding objective changes to the EHR following ORA implementation in pediatric contexts

**Box 1:** Overview of biomedical ethical norms for clinical practice

^a^We recommend that research investigates adolescents’ and parents’ experiences in different contexts and settings. We envisage that a variety of methodologies should be applied to tackle these research questions including mixed methods survey research, natural language processing, and other techniques aimed at understanding objective changes to the EHR following ORA implementation in paediatric contexts.
**Autonomy** refers to the right of patients to make informed decisions about their own medical care without coercion or undue influence from HCPs or others [15]. This concept emphasizes the importance of respecting a patient’s access to understandable health information and their personal values, preferences, and choices regarding their treatment options, even if those choices differ from the recommendations of HCPs; see also Box 2.
**Nonmaleficence** refer to the obligation of HCPs to prevent harm, whether through action or inaction, and to carefully weigh the risks and benefits of any treatment or intervention [15]. In practice, nonmaleficence involves ensuring that the potential harm does not outweigh its potential benefits.
**Beneficence refers to** HCPs’ obligation to promote beneficial health outcomes and promote patient wellbeing [15].
**Confidentiality** refers to HCPs’ responsibility to share information responsibly, and informational privacy refers to the patient’s right to security of their health data [15].

## Results

### Respect for persons’ autonomy and self-determination

While respect for adult patient autonomy is comparatively uncontroversial, especially since the post-World War II era, resolving how to protect or strengthen autonomy among persons during childhood and adolescence, and among patients with cognitive decline or cognitive vulnerabilities is more challenging. When it comes to adolescents, the dominant view is that with age, adolescents should be provided with increasing respect for their autonomy, and right to self-determination in their health decisions. Although there is agreement that young minors lack capacity to make medical decisions, it is also agreed that the growing autonomy of the young person should be supported via measures such as ‘assent’ (see Box 2); however, there is some disagreement about how and when to implement assent and consent.

**Box 2:** Background on respect for autonomy among adolescents

HCPs have a duty to respect patient autonomy. However, the extent to which respect for patient autonomy applies to children and adolescent patients is debated. This is due to the underdeveloped capacities of young people, and their developing ability to understand medical information and weigh it up effectively to make fully informed decisions about their health. How HCPs can support and facilitate the development of autonomy among younger people in medical contexts is also debated.
In paediatrics, obtaining assent from a child involves explaining a medical procedure or treatment in an age-appropriate manner and ensuring that the child understands and agrees to the plan, even if parental consent is also required. This process respects the child’s developing autonomy and involvement in their own healthcare decisions.
In clinical ethics, there is ongoing debate about how to implement ‘assent’ among paediatric patients, with a lack of consensus on appropriate guidelines. The American Academy of Pediatrics suggests that “active agreement” may be suitable from the intellectual age of 7, with “assent” applicable between ages 8 to 14, though it ultimately leaves the decision to the discretion of healthcare providers. This flexibility allows for contextual judgments [19], but excessive interpretative latitude might lead to inconsistencies in how standards are applied, potentially neglecting the child’s role in decision-making. Some medical ethicists argue against a single predetermined age for assent, advocating instead for decisions based on factors such as the complexity of the medical intervention, the child’s maturity, and their familiarity with clinical settings [20]. Some evidence-based approaches suggest that children over 12 may have the capacity to understand and reason about medical information, though this is complicated by evidence indicating that adolescents may have underdeveloped executive functioning, making them more prone to underestimate risks [21]. Adapting the MacArthur Competence Assessment Tool for Clinical Research– a validated instrument for gauging adult competence to participate in research– Hein et al. [22] investigated the correlation between age and children’s capacity to provide consent, and found that children over the age of 12 are capable of understanding and reasoning about medical disclosures, concluding that currently established ethical and legal age limits for informed consent are unreasonably low.

#### Knowledge and recall

Remembering information from face-to-face visits or consultations can be overwhelming, particularly for adolescents who can be distracted or absent-minded. More informed parents may be better equipped to provide care for and educate their child about their condition. Providing information through ORA, could improve health literacy and treatment adherence among adolescents and parents.

##### Adolescents

As adolescents gradually develop greater autonomy, ORA can serve as a tool to support and foster this progression, while maintaining an appropriate balance with the guidance provided by parents and HCPs. For example, ORA can be a memory aid for patients, considering experts’ estimate of 40–80% of information being misremembered or forgotten [23]. Adolescents aged 12–18 years old without ORA experience anticipate that reading their EHRs would allow them to gain knowledge about their health [24] and remember visits better [25]. One adolescent claimed the benefit of recall being especially valuable to teenagers, who might not listen fully or not completely understand everything said during an appointment [26]. Especially adolescents with a chronic illness appear to benefit from increased health-related knowledge [27], and adolescent portal users with cancer report improved memory recall and being able to ask informed questions and reflect more on their health [28].

Still, if adolescents lack interest or are unable to understand the information in their EHRs in practice, ORA does not contribute to enhancing their autonomy. In a recent study, 45% (23/51) of adolescents described a lack of interest in using a portal [27]. Reported barriers to use included difficult or intimidating information, and feeling that the portal was intended for parents and adults rather than for adolescents. Lack of interest and time has been observed among adolescents without a chronic illness [27], and observational studies have identified low use among adolescents [29]. However, patients who are given access earlier show higher rates of use during late adolescence [30]. It should be noted that adolescents, with and without chronic illness, report curiosity about ORA [27] and this is the most common reason for accessing EHRs for this age group [31]. Furthermore, adolescents without access to their EHRs expressed worry about not being able to understand and appropriately interpret the information in the EHRs [32, 33]. In a study of 69 young patients aged 12–20, approximately two thirds demonstrated note comprehension that agreed with physicians’ interpretations [34]. Among 20 psychiatric patients aged 12–17 who did read their records, the average self-reported understanding of a medical note at 8.5 (of 10) on average, however half reported not understanding the discharge criteria [35]. The empirical findings support the conclusions by Hein et al. [22], that children aged 12 and older are capable of understanding and reasoning about medical disclosures, as demonstrated by high self-reported comprehension scores and alignment with physicians’ interpretations in many cases. However, the noted gaps in understanding specific details, such as discharge criteria, align with Hein et al.‘s observation that adolescents may require additional support to fully engage with complex medical information.

##### Parents

For parents, ORA can serve as a valuable tool in fulfilling their parental responsibilities, focusing on their role as caregivers. Strengthening parental autonomy to make informed decisions may improve adolescent health outcomes related to attendance and treatment adherence. However, it is important to consider adolescents’ and parents’ capacity to understand any such information housed in the EHR.

To date, multiple studies of parents reading their child’s records have identified self-reported increased knowledge and understanding of their child’s health [5, 36–42]. In a focus group study including eight parents with experience caring for a hospitalized child, parents anticipated that ORA may facilitate better advocacy for their child [41]. However, they worried about not understanding some medical jargon used by clinicians. In studies of parents with access to their child’s EHR, some have reported being able to guide their adolescent child, teach them about their illness, and being better able to track the child’s illness [27]. Several studies have observed parents to appreciate having access to test results [43, 44]. Dutch parents experienced increased ownership and increased capability from ORA [45], while doubting younger adolescents’ capacity to deal with sensitive information in their EHR.

##### HCPs

In a US focus group study, clinicians in adolescent care anticipated that ORA can provide adolescents and parents with increased knowledge and enhanced recall of visit events [24]. Mental health HCPs believed that reading the EHR could improve adolescents’ understanding of their condition [8]. In a survey study conducted in a neonatal intensive care setting [46], most HCPs (76%) anticipated that parental ORA would increase parents’ knowledge, though 100% believed that there may be EHR information that parents would not understand. Among HCPs with ORA experience, oncology HCPs reported that sick adolescents tend to depend on parents and that ORA facilitates parental care [47], for example in managing medications and viewing lab results. However, HCPs had concerns about young adolescents (aged 12) dealing with confidential information in the EHR pertaining to their parents or families [45].

While existing work indicates adequate comprehension among some adolescent patient groups, findings call for further investigation of adolescents’ and parents’ comprehension of patient portal information. We recommend further work exploring interest in accessing a patient portal among adolescents who are informed about its functions. Lastly, validated measures could be applied to explore perceived improvement of knowledge and recall among adolescents and parents from reading EHRs. These aspects should be explored among adolescents with and without serious health issues.

#### Improved transition to adult care

By demonstrating trust in the patient, including the parent, as a responsible care partner and allowing more time for reflection on shared information, ORA could enhance both adolescent and parental agency and potentially contribute to strengthened respect for parental preferences as well as those of the maturing young person.

##### Adolescents

Allowing adolescents to assent to parental access to their ORA can enhance their engagement in their own healthcare by encouraging open communication, promoting shared decision-making, and fostering a sense of responsibility for their health. This process also provides an opportunity for adolescents to better understand the purpose and benefits of ORA. Furthermore, beyond individual empowerment, ORA might support adolescents in navigating the social relationships and contexts that significantly influence healthcare decision-making, preparing them for greater autonomy in managing their health. Though research on adolescents’ ORA experiences is limited, adolescent portal users with cancer anticipated that ORA could support the transition from paediatric to adult care [28], and an adolescent with haemophilia suggested that the records could be jointly managed by themselves and their parents during the transition to adult care [48]. Moreover, a Dutch quantitative study found that ORA strengthened a growing sense of autonomy among adolescents [45], as it enhanced their sense of ownership and facilitated a stronger capacity to manage their care according to their level of motivation to do so.

##### Parents

Parents with ORA experience believed that viewing notes could contribute to empowering adolescents in their own care and increase adolescent agency in the transition to adult care [24, 41]. Parents have reported appreciating not having to bother clinicians [36, 37, 40]. Furthermore, caregivers of children with cancer saw an advantage in ORA as a way for their child to go back and better understand their care in the future [40]. In alignment, parents of children with cancer have reported believing that the ORA would be useful when transitioning into adult care or another care provider [28].

##### HCPs

Clinicians without ORA experience also foresee that the tool could empower adolescents and support the transition to adult care [24], and within mental healthcare, HCPs anticipated that a newly introduced patient portal could better engage adolescent patients in their own care [8]. With ORA experience, one clinician reported believing that ORA can empower adolescents in their own care [47]. However, some oncology HCPs perceive that adolescents lack interest and capacity to read their EHRs, as the care responsibility is largely put on the parents [47]. Dutch clinicians observed increased parental ownership from ORA, as parents were providing feedback in the EHR on reports and planned appointments (making edits and additions were possible in the portal) [45].

So far, preliminary empirical research suggests stakeholders anticipate that adolescents’ transition to adulthood can be supported by reading their EHRs. In this way, ORA could contribute to the principle of autonomy. However, additional work is necessary to explore how the transition is supported, in what contexts and for which patients and their parents. It will also be necessary to engage adolescents and parents in dialogue with HCPs to inform all parties about proxy access, confidentiality, and responsible use of confidential data. In addition, in some circumstances - such as sexual abuse, for example - private communication between HCP and adolescent patients is essential.

### Nonmaleficence

Clinicians have a duty to ‘first, do no harm’, and how ORA intersects with nonmaleficence has been the subject of growing ethical and empirical explorations, particularly in relation to adult patients with mental health conditions, or cancer [49–51]. Once again, however, few studies have examined child or adolescent health. Below we discuss the possible varieties of harm that may arise from ORA (please also see *Privacy and Confidentiality*).

#### Adolescent and parental anxiety

Health anxiety is the excessive worry about one’s symptoms or the risk of developing a serious illness. Immediate access to worrisome or confusing information in the EHR, without face-to-face explanation from a HCP, could lead to increased anxiety among adolescents and parents [24, 42]. A risk of extreme anxiety among adolescents is intentional self-harm. Potential harm in allowing adult patients in mental health to read their records has been stressed by HCPs, and there is risk that adolescents present with even greater vulnerability, due to undergoing cognitive and emotional development. Increased prevalence of self-harm among adolescents has been observed in Europe [52] and Asia [53] during the last decade.

##### Adolescents

Empirical research has found that adolescents with no ORA experience believe that some portal content could lead to increased distress, and that some would prefer receiving bad news from parents [27]. In a focus group study, adolescents described ways that ORA may lead to negative emotions [24], such as by feeling judged or offended by mention of sensitive and stigmatizing information (for example pertaining to weight, sexuality, or mental health). No research has explored expectations or experiences related to self-harm because of ORA. The few studies focused on adolescents in mental health care have been quantitative and focused on literacy and satisfaction [35] or interest in patient portals [54].

##### Parents

Parents of adolescents with cancer have expressed concerns about adolescents being alone when receiving bad news in the EHRs [28]. Parents also worried about bad news being communicated in the EHR without face-to-face communication with HCPs [28, 41]. Parents without ORA have expressed concerns about whether worrying information could be challenging for adolescents struggling with anxiety [24], as well as stigmatizing information, such as mention of weight, particularly to female adolescents.

##### HCPs

Studies of HCPs, primarily with no experience of ORA, have expressed concern about adolescents and parents experiencing increased anxiety because of misinterpretation or accessing information, such as abnormal test results, on the portal without prior explanation from a HCP [5, 24]. In a U.S. study, oncology clinicians reported experiencing work interruption and an increased workload from having to provide support and reassurance to families who received bad news via the EHR, and consequently putting effort into providing anticipatory guidance about portal use to families [47]. In Norway, where adolescents have access from age 16, Norwegian mental health clinicians worried about harm to the adolescents’ mental health from reading the EHR [8].

While concerns about patient anxiety are reported by HCPs, adolescents are an especially vulnerable population due to the ongoing development of brain functional systems [55]. Additional studies are needed to explore adolescent patients’ and parents’ experiences of distress and anxiety from accessing the adolescents’ EHRs, to examine effects on the duty of nonmaleficence. No studies have quantitatively explored the experiences of anxiety among adolescents or parents from accessing the EHR. Furthermore, and underexplored, it remains to be understood whether the potential for patient anxiety from reading EHRs might be reduced by increased empathy of language used in the EHR, for example using tools powered by generative artificial intelligence [56] which have been found to have particular strengths in offering cues and signatures of empathy in documentation [57].

#### Diagnostic accuracy

Accuracy of EHRs is critical to enable effective care. When adolescents and parents have access to notes, HCP may feel limited in their ability to document sensitive or worrisome information. Another risk is that adolescents and parents may be deterred from being completely honest with HCPs, leading to incomplete EHRs.

##### Adolescents and parents

Limited research has explored adolescents’ and parents’ expectations or experiences related to concerns about the accuracy of notes as a result of ORA. Adolescents and parents without experience of ORA worried that adolescents might not reveal sensitive information if they knew it would be visible to their parents [24, 41]. In a focus group study, parents of hospitalized children worried that HCPs may not be able to document their discussions due to knowing that parents will read notes [41].

##### HCPs

In international surveys, HCPs report changing their documentation with the knowledge that patients might read it [58, 59]. For example, a survey of General Practitioners in England found that 72% (*n* = 289) said they will be/already are less candid in their documentation after ORA [60]. Growing research in paediatric settings suggests similar practices. Conceivably, the tendency for HCPs to ‘dumb down’ records, or omitting key clinical information could lead to patient harm [61]; it remains possible that the risks are even greater in scenarios when adolescents and parents are offered ORA. A third of HCPs (32%) in neonatal care expected that parental ORA would lead to improved documentation [46]. There is scarcely any research that has directly or indirectly explored this consideration.

Research has also described the difficulties experienced by HCPs of documenting in EHRs when the parent and HCP disagree on how to best meet the child’s medical needs [62]. Paediatric oncologists have described modifying their documentation to avoid insulting parents and avoiding documentation of sensitive information [47]. In a US study [42], attendings and interns at a US tertiary children’s hospital reported changing note content regarding cancer, mental health, substance abuse, obesity, or child neglect and/or abuse concerns (11% attendings and 15% interns). In a qualitative study, Norwegian mental HCPs believed that withholding EHR information could prevent emotional harm to young patients, while also reporting concerns about the consequences of omitting information [8].

These findings indicate that ORA may limit HCPs’ ability to fully document sensitive information about children in the EHR, out of concern for causing confusion, emotional distress, or damage relationships between patient, parents, and HCPs. Such concerns may compromise the clinical accuracy of notes and thereby compromise HCP autonomy. The original function of clinical documentation is to serve as an aide-mémoire and communication tool among clinicians, and it is unknown whether parental or adolescent access may devalue the utility of documentation for HCPs. Future research might thereby aim to investigate whether ORA in paediatric settings objectively interferes with records and diagnostic reasoning. Measuring the rate of diagnostic error in paediatric settings is challenging; however, use of patient surveys, retrospective case reviews, and clinical outcome measures could help examine rates of diagnostic accuracy following ORA [49, 63, 64].

### Beneficence

#### Treatment adherence

By providing immediate access to information about medication and appointments, ORA may improve adolescents’ adherence to treatment as well as facilitate parental support in this endeavour.

##### Adolescents

Adolescents with chronic illness reported that reading EHRs could reinforce the importance of taking medications and following HCPs’ recommendations, and being able to schedule appointments around social activities [27]. In a study of paediatric patients and parents by Bell et al., 37% (1,707/4,682) reported that reading notes helped with taking medications as prescribed and 55% (1,642/3,012) thought that notes helped them remember to go to appointments [65].

##### Parents

Parents of adolescents without chronic illness described how reviewing clinical notes might encourage teens to exercise or eat healthier if the recommendations came from physicians [27]. Qualitative studies have observed parents to appreciate being able to manage appointments [32], and observational studies have confirmed high access of appointment scheduling and review functions among parent proxy users [29, 44, 66]. A usage study of parents of children aged 0–11 found that 85% had accessed the appointment review feature, and 77% had accessed the medication feature [67].

##### HCPs

A psychiatry provider working in an adolescent inpatient setting reported that clinical note sharing helped inpatient counselling sessions and compliance for 8 of 20 patients aged 12 or older [35]. Furthermore, paediatric oncology clinicians reported that parental ORA can contribute to compliance and appointment attendance since adolescents can be unreliable [47].

Scarce work indicates that ORA for adolescents and parents can improve treatment adherence for adolescent patients, contributing positively to beneficence. Though observational studies find that appointment and medication management are highly used features among adolescents and parents, potential effects on treatment adherence and appointment attendance have not been studied. Therefore, future work is required to understand whether and how ORA influences adolescent patients’ adherence to treatment and medication, as well as in attending appointments.

#### Reduced anxiety

Waiting for laboratory test results or a potentially serious diagnosis can lead to high levels of anxiety. Immediate access to EHRs that are always accessible may alleviate such concerns among adolescents and parent caregivers.

##### Adolescents

Few studies have explored adolescents’ experiences with ORA, leaving limited evidence regarding positive effects on adolescents’ emotional state. Qualitative work has indicated that adolescents with chronic illness reading their EHRs report that quick access to test results reduces their anxiety [27, 28].

##### Parents

Qualitative work indicates that parents of children with chronic illness experience reduced anxiety from having access to their children’s records [68], and parents of children with cancer with ORA feel a sense of control and reassurance [40]. Of parents reading their hospitalized child’s EHR, 91% reported an increased sense of control [42].

##### HCPs

Of HCPs in a neonatal intensive care unit, 62% anticipated that parents may feel more reassured in their child’s care by reading their child’s EHR [46]. Qualitative findings indicate that HCPs perceived parents to feel in control from accessing daily progress notes for their hospitalized child [42].

Qualitative findings thus suggest that ORA might lead to positive influence on adolescents’ and parents’ wellbeing. However, the contrasting risk of increased anxiety from receiving bad news without face-to-face communication has been described under Nonmaleficence. Future research is needed to quantitatively assess adolescents’ and parents’ emotional experiences from reading the EHRs and potential effects on health outcomes, specifically among adolescents with serious health issues.

#### Perceiving errors

When documenting in the EHR, HCPs can make mistakes that can compromise patient safety. In one study 26% of HCPs anticipated before ORA implementation that patients would find an error in their EHR [69]. In a study conducted among General Practitioners in England, 60% (240/400) believed a majority of patients would find significant errors in their EHR [60]. Providing patients with ORA might better facilitate identification of errors and thereby help to prevent medical errors and possible harms that may result.

##### Adolescents

Adolescents with cancer and blood disorder who were using a patient portal appreciated being able to check note accuracy after visits [28]. A survey study by the authors found that 28% (60/218) of adolescent participants had identified an error in their EHR, of whom 64% (55/86) did not act upon finding an error [70].

##### Parents

Among parents, the benefit of error detection was both reported by those with experiences of accessing their child’s records [26, 34, 36, 41] and anticipated by those without [36]. In an observational study, Lam et al. [71] reported that 17% (1,434/8,648) of adult patients and parent proxy users identified an error in the EHR, whereby half of parents (342/614, 56%) who identified errors in their child’s records reported it to their healthcare provider. However, the definition of EHR errors is subjective and could include spelling mistakes, typing errors, or originate in the patient disagreeing with the HCP’s views. There is yet no evidence of the clinical importance of patient-reported errors. Notably, in the study by Lam et al. [71], 22% of those who reported finding a perceived serious error did not report it, due to uncertainty on whether it was in fact serious.

##### HCPs

In regards to HCPs, Chung et al. [46] reported that about half of HCPs in a neonatal intensive care unit believed that parents accessing their child’s EHR may help identify incorrect (70/133, 53%) and missing (84/133, 63%) information. In another study of HCPs’ experiences of adolescent patients and parents using an inpatient ORA tablet application, 3% of questions from patients and parents concerned errors [72].

There may be an important role for parents, and/or adolescents to assist with closing the feedback loop on care, via improving EHR accuracy via error reporting. Future research is required to explore adolescent patients’ and parents’ experiences of identifying errors in the EHR, whether these were validated as such, and any subsequent actions taken to rectify purported inaccuracies. Such insight will be crucial to determine whether, on balance, ORA can strengthen, the duty of nonmaleficence in healthcare.

### Privacy and confidentiality

#### Access breaches

EHRs contain personal and confidential data. Non-consensual access of parents or others can harm patients’ trust and willingness to seek care, especially for adolescents who tend to harbour an increasing desire for privacy. Furthermore, parents may be deterred from disclosing confidential information if it is visible in adolescents’ EHRs.

##### Adolescents

Amongst the limited work that has focusing on adolescents’ views on ORA, confidentiality concerns about parents accessing information in their records have primarily been reported by adolescents who have not accessed their EHR [32, 33]. For example, American adolescents aged 16–18 in primary care wished for billing information to not include details about received care [32]. In an observational study of a US health institute evaluating its ORA implementation for minors and parents, no confidentiality complaints from minors were reported [67]. An interview study with 23 adolescents with cancer or blood disorders aged 13–17 revealed no confidentiality concerns, however it is possible that these adolescents have a unique perspective on confidentiality [28]. Adolescents reading their EHR have stated risks in that some teens may overshare medical information in person, via social media, or sharing log-in credentials with friends [27]. In a study conducted by the authors on Swedish adolescent patient portal users aged 15–19 [70], 80% (164/218) of respondents perceived that their EHR maintained a high level of security, while 7% (15) reported that someone had accessed their records without their consent. Furthermore, 41% wanted to be able to manage who could access their EHR. Adolescents self-reporting poor health were more likely to desire the ability to manage who could access their EHR (*p* =.009). Evidence has identified that access to patient portals in the name of adolescent patients has been obtained notably by the parent [73], which underscores the complexity of balancing adolescent autonomy with parental involvement.

An additional challenge arises when categories of personal health information in adolescents’ records are subject to different confidentiality rules. For example, in the US, adolescents may consent to and control access to data on issues such as reproductive health, mental health, and substance abuse, while other issues, such as routine medical care, remain under parental control. When these types of information are housed within a single record, allowing blanket access risks violating the adolescent’s right to confidentiality, whereas restricting access may deprive parents of their legitimate right to information. Also, we observed in a survey study that what constitutes sensitive information for adolescents can vary based on individual factors [70]. The current lack of mature data segmentation tools exacerbates this issue, imposing a significant burden on clinicians tasked with navigating these conflicting obligations.

##### Parents

Parents with no experience of ORA express concerns about billing of confidential services causing privacy breaches [32]. Even if parents are restricted from access to their adolescent child’s account, there is a risk of parents accessing the adolescents through their account, with or without consent. Such incidents have been observed in studies using natural language processing methods, one finding that half of messages sent from adolescents’ accounts were written by parents [73], and that parents’ contact information was often associated with the adolescents’ account [74]. With respect to adolescents having their own access, parents were concerned that adolescents might use the portal inappropriately and would need education [27, 32, 48]. Among 83 parents of adolescents aged 13–17 at a juvenile detention facility, 63% had no privacy concerns about their adolescent child accessing their EHR online, while reported concerns included that the portal might be hacked by a malicious third-party [75]. There are additional risks, such as a situation of intimate partner violence described in a case study, where information from the mother’s EHR automatically populated a new-born child’s EHR and became accessible to the father [76].

##### HCPs

Echoing the views of adolescents and parents, HCPs’ main concern related to ORA is being able to preserve adolescents’ and parents’ confidentiality [18]. Among clinicians with ORA experience, 40% of 212 clinicians were not at all confident that their EHR maintained privacy for minors, with 82% expressing concerns about maintaining confidentiality [4]. HCPs in gynaecology and psychiatry had concerns that adolescents may not seek care if unsure about confidentiality [77, 78]. In US study of 26 paediatric health care providers with experience of sharing EHRs with adolescents, Stablein et al. [79] reported that confidentiality concerns affected documentation practices, in that all HCPs involved in the child’s care will not be aware of what information in the record is private from parents versus what the parent needs to know. Bourgeois et al. [62] have described situations where ORA causes ethical dilemmas for HCPs, for example, when the adolescent or parent discloses sensitive information to the HCP that they wish to conceal from the other. As a result of such difficulties, some have proposed that portal access for adolescents should be limited until the privacy functionality is more secure [80].

It should be noted that although research so far has not identified any major confidentiality concerns among adolescents with experience of ORA, those most negatively affected by breaches to confidentiality (e.g. controlling parent such as honour-based contexts, minors in ongoing custody battles, victims of abuse) likely constitute a minority and may be difficult to reach. Further research must thus examine the dangers potentially posed to these minors by ORA and how they can be mitigated.

Findings indicate that parents access their adolescents’ accounts to manage their care after having lost their own access and HCPs hold extensive concern about the possibilities to document information securely. Medical professionals favoured customizable controls of information display for both parents and adolescents [80]. Attempts have been made to enable portal functionality that aid HCPs in protecting adolescents’ EHR information. Kaufman et al. [81] developed a confidential shared teen sexual EHR section for managing teen sexual history, observing documentation of sexual activity in 72% of notes. In the Netherlands, researchers developed a patient portal where adolescents can decide to conceal information from their parents [82]. While these are important efforts, further work is needed to examine how the confidentiality of adolescent patients and parents in EHRs can be ensured. Researchers and HCPs have suggested customizable controls of information display for both parents and adolescents [80], emphasized the need for HCPs to be able to label information as confidential [78, 83, 84], and for enabling adolescents to restrict parental access [85]. In Finland, where parental access depends on minors’ consent, data shows that 96% of minors chose to disclose information to guardians [30]. Still, further examination of stakeholders’ experiences of approaches allowing adolescents’ control of granting and blocking access to their EHRs is warranted.

#### Trust

Trust in medicine, particularly between adolescents and doctors, is a foundational element that ensures effective communication. It requires a delicate balance of respecting the adolescent’s autonomy while safeguarding their best interests, fostering an environment where the adolescent feels safe, understood, and valued. Similarly, trust between parents and doctors is essential in paediatric care, as it fosters collaborative decision-making, can better ensure that the child’s health needs are met, and strengthen a partnership necessary for delivering effective and compassionate care.

##### Adolescents

Adolescents aged 12–18 with no ORA experience anticipated that access to notes can create a feeling of being understood, improve trust for the HCP and strengthen the relationship [24]. A US study set in inpatient psychiatric care found adolescent patients’ trust in their HCP to increase or remain the same from ORA [35]. In a survey study by the authors, around two-thirds of Swedish adolescent patient portal users reported experiencing more trust in their HCP (143/218, 66%) and better communication with healthcare from reading their EHR (145/218, 67%) [70]. However, adolescent patient portal users aged 12–17 (with or without chronic illness) have stated that the tone used by HCPs in clinical notes can seem judgmental [27]. Survey research by the authors [86] has found that 26% (57/218) of national patient portal users in Sweden aged 15–19 had been offended by reading their EHRs, where the most commonly reported reason was disrespect.

##### Parsssents

Several studies have found that parents experience enhanced communication and partnership with providers from ORA [5, 36–38, 41, 42, 65, 68]. In a large-scale US survey of adult patients and parents [65], 42% (3,752/10,252) reported trusting the healthcare provider more from reading notes. Adolescents and parents accessing the EHRs worried about a strain on the relationship between child and parent if the child found out information that the parent was trying to conceal [27]. Parents in a Dutch study reported that the transparency of ORA led to enhanced trust in HCPs, as well as a more equal relationship and collaboration between them [45].

##### HCPs

Paediatric HCPs are, similarly to adolescents and parents, divided on the impact of ORA on trust. Qualitative studies have found that expectations of improved patient and parent trust among HCPs in a US children’s hospital [24] and among Norwegian mental health HCPs [8]. However, while 53 interviewed US oncology clinicians confirmed that ORA transparency strengthened their connection with parents, they also described that portal access could impede the clinical relationship by upsetting parents who identify inaccurate, insulting, or unexpected documentation in the EHR [47]. Concerns about upsetting parents due to disagreement were also reported by mental health HCPs [8].

Findings demonstrate that access to adolescents’ EHRs might increase young patients’ and parents’ trust with their healthcare provider, by being transparent about clinical work. However, there is also a risk that ORA may affect the clinical relationship negatively, as well as complicate the relationship between patient and parent. It is unknown under what clinical contexts, and for which patients, ORA can positively and negatively affect trust among adolescents and parents, and among both respective groups and their HCPs. For example, attention should be given to transgender healthcare, as an oncology clinician stated that misgendering in the EHR can hurt the patient’s trust [87], and Swedish adolescents have cited being misgendered in the EHR as a reason for being offended [86].

## Discussion

Concerns about harm have raised ethical questions about whether ORA should be implemented in paediatric settings. A lack of guidance and principles has led to global variability in access where some adolescents and parents are fully prevented access. It is necessary to evaluate whether expectations about benefits and harm are aligned with actual experiences. In this study, we used biomedical ethical principles to discuss ORA for adolescents and parents. Findings indicate that access to EHRs for adolescents and parents has the potential to contribute positively to adolescent health by, for example, engaging adolescents early in their care, supporting adolescent education, and facilitating parental support, which is critical particularly for adolescents with serious health issues. However, research is limited on adolescents’ experiences, and differences across settings have not been sufficiently examined. 

Confidentiality concerns persistently reported by HCPs remain notably unreflected in research on adolescents’ experiences. To better understand and mitigate HCPs’ worries, an essential inquest for future work is to investigate experiences of minors and parents in circumstances where EHR breaches may pose a risk of serious and imminent harm. Another area of uncertainty concerns adolescents’ and parents’ understanding of EHRs and adolescents’ experiences of feeling judged. HCPs hold concerns about patient anxiety from not understanding information, or being offended by what they read. These concerns, in addition to confidentiality queries, lead to HCPs changing and omitting information in the EHR, leading to a perceived decreased quality. Future work is necessary to investigate whether note writing can be improved to increase adolescents’ and parents’ literacy and feeling of being understood, rather than judged. The interplay between adolescent assent, which respects their developing autonomy, and parental consent, which ensures parental oversight, presents a complex ethical dynamic. Research is needed to explore how the process of obtaining adolescent assent influences their engagement in healthcare, the extent to which it supports their autonomy, and how it can be effectively balanced with parental involvement to promote both trust and collaborative decision-making.

## Conclusions and recommendations

Much uncertainty remains in regards to adolescents and parents accessing adolescents’ EHRs. At a minimum, patient portals must contain functionality that allow paediatric HCPs to protect adolescents’ and parents’ privacy, and HCPs require training to use such features. Also, a dialogue between minor patient, parent, and HCPs about patient portals is needed to increase understanding about confidentiality and mitigate concerns that could affect adolescents’ decisions to seek care. Encouragement of HCPs can lead to increased use among adolescents [31] and some researchers even advocate that HCPs in adolescent healthcare ought to adopt an autonomy-supportive attitude [45]. A critical factor in the design and implementation of ORA systems is the incorporation of assent processes that respect and support adolescents’ developing autonomy. This involves creating mechanisms that allow adolescents to actively participate in decisions about their health data, fostering a sense of agency and responsibility. Respecting their assent means acknowledging their capacity to contribute to healthcare decisions in a manner appropriate to their maturity level. It also requires ensuring that they are adequately informed about the implications of their choices, including how parental access to their records may impact their privacy and decision-making. Thoughtfully designed assent processes not only reinforce respect for young persons including of their developing autonomy but also encourage open dialogue between adolescents, parents, and healthcare providers, ultimately promoting trust and collaboration in the care process. For example, with regard to parental proxy access, we suggest that adolescents should be consulted throughout adolescence, to ensure that parental insight is in line with current needs, preferences, and other life circumstances.

In conclusion: addressing ethical dilemmas and advancing ORA in paediatric care will require a dedicated, ongoing effort from healthcare providers in reevaluating the role of clinical documentation [88]. This involves discussions on maintaining accuracy in medical records while enhancing the use of notes as a communication tool with both patients and their families. Paediatric HCPs will need specialized training and guidance on how to effectively use clinical notes to improve patient care. Families and caregivers will also need clear guidance on how to interpret these notes, including how to bring up any concerns with the healthcare team.

## Data Availability

The data that support the findings of this study are available from published, peer-reviewed publications, and references are provided. Data should however be available from the authors upon reasonable request.

## Abbreviations

EHR: Electronic health records
EU: European Union
HCP: Healthcare professional
ORA: Online record access
UK: United Kingdom
US: United States
